# Host Heterogeneous Ribonucleoprotein K (hnRNP K) as a Potential Target to Suppress Hepatitis B Virus Replication

**DOI:** 10.1371/journal.pmed.0020163

**Published:** 2005-07-26

**Authors:** Lisa F. P Ng, Marieta Chan, Soh-Ha Chan, Paul Chung-Pui Cheng, Eastwood Hon-Chiu Leung, Wei-Ning Chen, Ee-Chee Ren

**Affiliations:** 1 Genome Institute of Singapore, Singapore,; **2** Department of Microbiology, Faculty of Medicine, National University of Singapore, Singapore,; **3** School of Biological Sciences, Nanyang Technological University, Singapore; Royal Free and University College Medical SchoolUnited Kingdom

## Abstract

**Background:**

Hepatitis B virus (HBV) infection results in complications such as cirrhosis and hepatocellular carcinoma. Suppressing viral replication in chronic HBV carriers is an effective approach to controlling disease progression. Although antiviral compounds are available, we aimed to identify host factors that have a significant effect on viral replication efficiency.

**Methods and Findings:**

We studied a group of hepatitis B carriers by associating serum viral load with their respective HBV genomes, and observed a significant association between high patient serum viral load with a natural sequence variant within the HBV enhancer II (Enh II) regulatory region at position 1752. Using a viral fragment as an affinity binding probe, we isolated a host DNA-binding protein belonging to the class of heterogeneous nuclear ribonucleoproteins—hnRNP K—that binds to and modulates the replicative efficiency of HBV. In cell transfection studies, overexpression of hnRNP K augmented HBV replication, while gene silencing of endogenous hnRNP K carried out by small interfering RNAs resulted in a significant reduction of HBV viral load.

**Conclusion:**

The evidence presented in this study describes a wider role for hnRNP K beyond maintenance of host cellular functions and may represent a novel target for pharmacologic intervention of HBV replication.

## Abstract 2

A host protein -hnRNP K- may have a crucial role in determining how well the Hepatitis B virus replicates in humans.

## Introduction

Hepatitis B virus (HBV) infection is a serious public health problem in many parts of Asia and Africa that results in complications such as cirrhosis and cancer of the liver [1]. Globally, despite effective vaccination against HBV and available antiviral treatments [2–7], there remain an estimated 350 million hepatitis B carriers with a lifetime risk for developing cirrhosis and hepatocellular carcinoma, as many individuals infected during infancy remain infectious carriers for decades. Although the association between HBV infection and the development of liver cancer has stirred profound interest in the development of therapeutics for both the prevention of HBV infection and the clearance of virus from chronic infection, HBV continues to be a pathogen of significant importance. Current antiviral therapies for HBV carriers include treatment such as alpha-interferon or lamivudine, but the long-term resolution of disease is disappointing due to low seroconversion rates and the development of drug-resistant viral mutants [7].

HBV belongs to a family of viruses known as *Hepadnaviridae* and encodes only four genes in a highly compact viral genome: the surface gene *(S),* the core gene *(C),* the X gene *(X),* and the polymerase gene *(P).* Viral replication has been shown to occur via an RNA intermediate in the cytoplasm, but, unlike retroviruses, integration of HBV DNA into the host genome is not required. Despite a wealth of information on the virus itself and the possible role that host factors may play in the viral infectious life cycle [8–11], the direct relationship between chronically infected patients and host–pathogen interactions is poorly understood.

Host cellular factor heterogeneous nuclear ribonucleoproteins (hnRNPs) are known to be pre-mRNA binding proteins and shuttle intermediates between nucleus and cytoplasm. hnRNP K in particular has been implicated in diverse molecular and cellular functions, including nuclear–cytoplasmic shuttling [12], transcription, and translation [13]. Here, we aimed to study the importance of hnRNP K in the regulation of HBV viral replication.

Both over-expression and knockdown assays were designed to enhance HBV viral load and to silence the endogenous hnRNP K, respectively, within the cell to verify the effects of hnRNP K on HBV viral replication.

## Methods

### Serological Assays

Serum samples were aliquoted and stored at −20 °C until they were defrosted for testing. We examined serum HBV DNA levels by the Hybrid Capture II HBV DNA Assay (Digene, Gaithersburg, Maryland, United States).

### PCR and Sequencing of the Enh II/Pre-Core Region

DNA extracted from serum was amplified with primers 5′--3′ and 5′- GCTTGGAGGCTTGAACAG-3′ (94 °C for 2 min, followed by 35 cycles of 94 °C for 15 s, 50 °C for 30 s, and 72 °C for 1 min, and lastly followed by 72 °C for 7 min) with *Taq* DNA polymerase (Roche, Basel, Switzerland). Nested primers 5′- GTCAACGACCGACCTTGAGG-3′ and 5′- ACCAATTTATGCCTACAGCCTC-3′ were used in a second round of PCR (94 °C for 2 min, followed by 35 cycles of 94 °C for 15 s, 55 °C for 30 s, and 72 °C for 1 min, and lastly followed by 72 °C for 7 min). The size of this nested PCR product was 116 base pairs (corresponding to nucleotides 1682–1798) and was resolved in 1.5–2% agarose gels. PCR products were cleaned up using the Qiaquick PCR Purification Kit (Qiagen, Valencia, California, United States) and sequenced directly to confirm the identity of the products using the ABI prism dRodamine Terminator Cycle Sequencing Ready Reaction Kit (ABI-Prism 310; Applied Biosystems, Foster City, California, United States). Results of the sequences were aligned and compared.

### Construction of Plasmids

Plasmids pGL3-Control (a Luciferase plasmid with a simian virus 40 enhancer and promoter) and pGL3-Promoter (an enhancerless Luciferase plasmid with a simian virus 40 promoter upstream from the Luciferase gene) were obtained from Promega (Madison, Wisconsin, United States). Plasmid pGL3-Promo/A was constructed by amplifying the basic functional unit of Enh II by PCR using primers LucF (5′- GC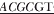 CAACGACCGACCTTGAGG-3′) and LucR (5′- GC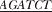 ACCAATTTATGCCTACAGCCTC-3′) comprising HBV nucleotide positions 1686–1801. The 131-base-pair PCR fragment was MluI/BglII-digested and ligated with MluI/BglII-digested pGL3-Promoter. The other mutant constructs were constructed using the Gene Editor Site-Directed in vitro Mutagenesis System (Promega) to introduce the HBV Enh II mutations at nucleotide position 1752. The first mutation was mutating nucleotide A to G (pGL3-Promo/G), the second was with nucleotide A to T (pGL3-Promo/T), and the third was with nucleotide A to C (pGL3-Promo/C). The sequences of the three mutant oligonucleotides were 5′- GGGGGAGGAG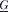 TTAGGTTAAA-3′, 5′- GGGGGAGGAG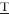 TTAGGTTAAA-3′, and 5′- GGGGGAGGAG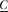 TTAGGTTAAA-3′, respectively. Constructs were sequenced for verification. hnRNP K “variant 2” and “variant 3” clones were constructed by cloning a 1.4-kb RT-PCR fragment coding for the hnRNP K from total RNA extracted from HepG2 cells. The EcoRI- and XhoI-digested PCR fragments were cloned into EcoRI- and XhoI-digested pcDNA 3.1 separately. Cloning primers for “variant 2” were 5′- TAAAAGGAATTCAATATGCAAACTGAACAG-3′ and 5′- CTAGTCCTCGAGTTAGAAAAACTTTCCAGA-3′, and cloning primers for “variant 3” were 5′- TAAAAGGAATTCAATATGCAAACTGAACAG-3′ and 5′- CTTGCACTCGAGTTAGAATCCTTCAACATC-3′. The HBV 1752A full-length replicative clone (genotype A) was constructed by using a HBV genome-containing pBR325 plasmid ( ATCC, Manassas, Virginia, United States) as a template. Primers were designed to amplify two fragments: 1–1900 and 1600–3215. The region 1600–1900 contained the core promoter and the overlapping transcription termination region [14] (Figure S1). In-frame ligation of the two fragments using the internal EcoRI (1/3215) ensures continuous viral open reading frames were cloned into the NruI site in pcDNA 3.1, resulting in the replicative construct. Viral transcription was under its own promoter control as the viral insert was cloned at the NruI site, located upstream of the plasmid's CMV promoter [15]. The 1752ΔG, 1752ΔT, and 1752ΔC full-length replicative clones were constructed as described for the 1752A. The first fragment, 1600–3215, was generated as a PCR product from the HBV-pBR325 plasmid and cloned into pcDNA 3.1. The 1752G, 1752T, and 1752C mutations were each generated separately in the first fragment by the Quick-Change site-directed mutagenesis kit (Stratagene, La Jolla, California, United States). Sequencing was done for verification of the constructs. The second fragment, comprised of nucleotides 1–1900 of the HBV insert, was generated by PCR from HBV-pBR325 and cloned downstream of the first fragment in pcDNA 3.1.

### Cells

Four cell lines—HCCM, HepG2, PLC/PRF/5, and Sk-Hep-1—derived from human hepatocellular carcinomas were used in this study. HCCM and PLC/PRF/5 contained copies of the integrated HBV genome, while HepG2 and Sk-Hep-1 were obtained from patients with no history of HBV infection and HBV genome integration [16–18]. Cells were cultured and maintained in complete Dulbecco's modified Eagle's medium (Invitrogen, Carlsbad, California, United States) and supplemented with 10% fetal bovine serum (Hyclone, South Logan, Utah, United States) at 37 °C in humidified 5% carbon dioxide.

### Transient Transfection Assays

Cells were plated at an average seeding density of 1 × 10^6^ cells per well in 35-mm tissue culture dishes and transfected with Lipofectamine 2000 (Invitrogen) according to manufacturer's instructions. Briefly, 2 μg of plasmid DNA was used for each transfection mix and added dropwise onto the cells. After incubation for 48 h at 37 °C, the cells were subsequently harvested, followed by DNA isolation (Qiagen). HBV viral loads were measured by real-time PCR using the RealArt HBV LC PCR Kit (Artus GmbH, Hamburg, Germany) according to manufacturer's instructions in the LightCycler Instrument (Roche). Experiments were done in duplicate.

### Luciferase Assays

For the luciferase assays, 3 μg of plasmid DNA together with 1 μg of control/promoter luciferase plasmid-DNA was used for each transfection mix, and, after incubation for 48 h at 37 °C, cells were harvested with Cell Culture Lysis Reagent (CCLR; Promega). Next, 20 μl of cell lysates was mixed with 100 μl of Luciferase Assay Reagent (Promega), and luciferase activity was measured as relative light units determined with a Turner 20/20 luminometer (Promega). Relative luciferase activity was expressed as fold increase over vector without the enhancer element. Experiments were performed in triplicate.

### Preparation of Nuclear Protein Extracts and Gel-Shift Assays

Cultures were trypsinized, rinsed twice with ice-cold 1× phosphate-buffered saline, and incubated on ice for 10 min with 5× original packed cell volume of Buffer A (10 mM *N*-2-hydroxyethylpiperazine-*N*′-ethane sulfonic acid [HEPES] buffer [pH 7.9], 1.5 mM magnesium chloride, 10 mM potassium chloride, and 1 mM dithiothreitol [DTT]). After centrifugation at 1,000 rpm for 3 min at 4 °C, cells were re-suspended in 2× original packed cell volume of Buffer A and homogenized in a Dounce homogenizer with an S pestle with 10 strokes. Nuclei fractions were sedimented by a 10-min centrifugation at 2,500 rpm, re-suspended in 1.5× Buffer B (20 mM HEPES [pH 7.9], 0.2 mM ethylene diamine tetra-acetic acid [EDTA], 1.5 mM magnesium chloride, 420 mM sodium chloride, 0.5 mM DTT, and 25% glycerol) and treated with another 10 strokes of Dounce homogenizer. Cell suspensions were then transferred to microcentrifuge tubes and incubated for 30 min at 4 °C with gentle rotation. Nuclear debris was removed by centrifugation at 13,000 rpm for 40 min at 4 °C. The supernatant was dialyzed for 4 h against two changes of 200 ml Buffer C (20 mM HEPES [pH 7.9], 0.2 mM EDTA, 20 mM magnesium chloride, 20 mM potassium chloride_,_ 420 mM sodium chloride, 25% glycerol, 0.5 mM DTT, and 0.5 mM phenylmethylsulfonyl fluoride) at 4 °C. After dialysis, nuclear extracts were clarified by centrifugation at 13,000 rpm for 20 min. Nuclear extracts were then aliquoted and stored at −80 °C. Protein concentration was quantitated with the Protein Assay kit (Bio-Rad Laboratories, Hercules, California, United States) using acetylated bovine serum albumin as standard.

Binding reaction procedures were performed at 37 °C for 20 min in 20-μl reaction mixtures (10 mM Tris-HCl [pH 7.5], 50 mM sodium chloride, 1 mM EDTA, and 1 mM DTT) containing 10 μg of HepG2 nuclear extracts, 0.1–0.2 μg of non-specific competitor DNA poly (dI–dC) (Amersham Pharmacia Biotech, Piscataway, New Jersey, United States), and ^32^P-dATP end-labeled probe (1 × 10^4^ to 1 × 10^5^ cpm). Free DNA and DNA–protein complexes were resolved on 6% non-denaturing polyacrylamide gels. Gels were dried down under vacuum at 80 °C for 1 h before exposure to X-ray film (Biomax; Eastman Kodak, Rochester, New York, United States) at −80 °C. The sequences of the oligonucleotide probes (nucleotide changes are indicated) were:

Probe 1, AGACTGTGTGTTTAATGAGTGGGAGGAG;

Probe 2, AGTTGGGGGAGGAGATTAGGTTAAAGGT;

Probe 3, AGACTGTGTGTTTAATGCGTGGGAGGAG;

and Probe 4, AGTTGGGGGAGGAGGTTAGGTTAAAGGT.

### Affinity Capture of Host-Interacting Proteins, Two-Dimensional (2-D) Gel Electrophoresis, and Protein Identification

Nuclear protein extracts were obtained from HepG2 cells. HepG2 cells were harvested and rinsed twice with ice-cold Buffer A (0.15 M sodium chloride and 10 mM HEPES [pH 7.4]), and incubated on ice for 15 min with 5× original packed cell volume of Buffer B (0.33 M sucrose, 10 mM HEPES, 1 mM magnesium chloride, and 0.1% Triton X-100 [pH 7.4]). After centrifugation at 3,000 rpm for 5 min at 4 °C, the pellet was washed once with Buffer B and re-suspended gently on ice with 200 μl of Buffer C (0.45 M sodium chloride and 10 mM HEPES [pH 7.4]), with protease inhibitor cocktail [Sigma P8340]). The cell mixture was incubated for 15 min with gentle agitation followed by centrifugation at 13,000 rpm for 5 min. The supernatant was saved for DNA-binding proteins assay. Annealing of double-stranded oligonucleotides probes was done using 100 μl of deionized Milli Q water containing 1 nmol each of antisense probe and sense probe, which were labeled with biotin at the 3′ end, and 5′ end, respectively. Oligonucleotide mixture solutions were heated at 95 °C for 5 min and cooled slowly to room temperature. DNA-interacting proteins were captured as described. The oligonucleotide mixture was incubated with 5 mg Dynabeads M-280 streptavidin (Dynal Biotech, Oslo, Norway) at room temperature for 15 min in binding and washing buffer (5 mM Tris-HCl, 0.5 mM EDTA, and 1.0 M sodium chloride [pH 7.5]). The magnetic beads were then washed with binding and washing buffer and equilibrated with TGED buffer (20 mM Tris-HCl, 10% glycerol, 1 mM DTT, 0.01% Triton X-100, and 50 mM sodium chloride [pH 8.0]). 40 μg of extracted nuclear proteins was mixed 2:1 (w/w) with non-specific competitor DNA poly (dI–dC) (Amersham Biosciences, Little Chalfont, United Kingdom), and adjusted to 500 μl with TGED buffer. Nuclear proteins–poly (dI–dC) solution was added to the equilibrated magnetic beads–oligonucleotide probe at room temperature for 30 min. Unbound proteins were washed out with TGED buffer. Bound proteins were eluted with TGED buffer with 1 M sodium chloride. The same capturing and elution procedure was repeated another four times with new aliquots of nuclear proteins–poly (dI–dC) mixture. Eluted fractions were pooled and subjected to acetone precipitation. We performed 2-D gel electrophoresis according to the Amersham Biosciences protocol, with some modifications. Each sample containing acetone-precipitated proteins was made up to a volume of 350 μl with rehydration buffer (7 M urea, 2 M thiourea, 4% CHAPS, 0.5% IPG buffer [pH 3–10], and 1.0 mg of DTT). The mixture was mixed briefly by vortexing, and centrifuged at 13,000 rpm for 10 min. The supernatant was loaded to 18 cm (pH 3–10) nonlinear Immobiline DryStrips (Amersham Pharmacia Biotech), and rehydration was carried out actively at constant voltage (50 V) overnight. Isoelectric focusing was performed using IPGphor (Amersham Biosciences) at 20 °C in stepwise mode. Briefly, strips were focused at 500 V for 1 h, 2,000 V for 1 h, 5,000 V for 1 h, and 8,000 V for 12 h, with a total of 90 kVh accumulated. After isoelectric focusing, the IPG strips were incubated for 30 min in 15 ml of SDS equilibration buffer (50 mM Tris-HCl, 6 M urea, 30% glycerol, 2% SDS, 66 mM DTT, and a trace amount of bromophenol blue [pH 8.8]), followed by second incubation with the same buffer for 30 min with iodoacetamide (375 mg/15 ml) instead of DTT. 2-D vertical SDS-PAGE (Protein II XL, Bio-Rad Laboratories) was carried out using 10% gels at a constant voltage of 150 V for 6–8 h at 15 °C. Gels were stained with SilverQuest Silver Staining Kit (Invitrogen). Specific protein spots were cored out and de-stained according to manufacturer's instructions, after which the gel plug was dried and MS/MS analysis carried out by Proteomic Research Services (Ann Arbor, Michigan, United States).

#### In-gel digestion

Samples were subjected to trypsin digestion on a ProGest (Genomic Solutions, Ann Arbor, Michigan, United States) workstation as follows: Gel plugs were soaked in ammonium bicarbonate solution and reduced with DTT. Alkylation was performed by using iodoacetamide. Samples were incubated at 37 °C overnight in the presence of trypsin. Formic acid was added to stop the reaction.

Liquid chromatography/tandem mass spectrometry (LC/MS/MS) analyses: Peptides were cleaned up by using C18 ZipTips (Millipore, Billerica, Massachusetts, United States) and eluted with matrix (α-cyano-4-hydroxycinnamic acid) prepared in 60% acetonitrile and 0.2% TFA. 15 μl of eluent was processed on a 75-μm C18 column at a flow-rate of 20 nl/min. Eluent from the C18 column was fed into nano-LC/MS/MS on a Micromass Q-TOF2 mass spectrometer. MS/MS data were searched using a local copy of MASCOT search engine (Matrix Science, London, United Kingdom).

### RNA Interference

Small interfering RNA (siRNA) duplexes against hnRNP K were purchased from Dharmacon (SmartPool) (Lafayette, Colorado, United States), Qiagen (sequence: GCAGUAUUCU GGAAAGUUU), and Proligo (sequence: CUU GGGACUCU GCAAU AGATT) (Boulder, Colorado, United States). HepG2 cells were co-transfected in 24-well tissue culture plates with 1 μg of plasmid DNA (1752A replicative full-length clone) and the respective siRNA duplexes (2 μg) using 6 μl Lipofectamine 2000 (Invitrogen). After 48 h, the cells were collected and were RNA and DNA extracted. As controls, cells were also transfected with fluorescence-labeled non-silencing siRNA to monitor the transfection efficiencies. Transfections were performed in duplicate.

### Quantitation of hnRNP K Expression

Total RNA was isolated with RNeasy kit (Qiagen) according to the manufacturer's instructions. The concentration and purity of extracted RNA were determined by measuring the A_260_ and A_280_, and adjusted to 250 ng/μl, which was then used as a template for real-time RT-PCR. 2 μl of RNA was used in a one-step real-time RT-PCR reaction with the LightCycler RNA Master SYBR Green kit (Roche) using primers 5′- AGACCGTTACGACGGCATGGT-3′ and 5′- GATCGAAGCTCCCGACTCATG-3′, and performed according to manufacturer's instructions. Absolute quantitation of RNA was obtained by using standard curves created with in vitro–transcribed RNA by the T7 RiboMax Express in vitro transcription system (Promega). The concentration of purified transcribed RNA was measured by RiboGreen RNA quantitation reagent (Invitrogen). Serial dilutions of in vitro–transcribed RNA were prepared in duplicate.

### Statistical Analysis

A multi-alignment sequence analysis of the HBV genomes with ranked HBV viral load was first performed, and we compared the frequencies of the mutations at nucleotide position 1752 using Fisher's exact probability test.

## Results

### Correlation between High and Low Viral Loads in Patients at a Precise Location in the HBV Genome

**Figure 1:**
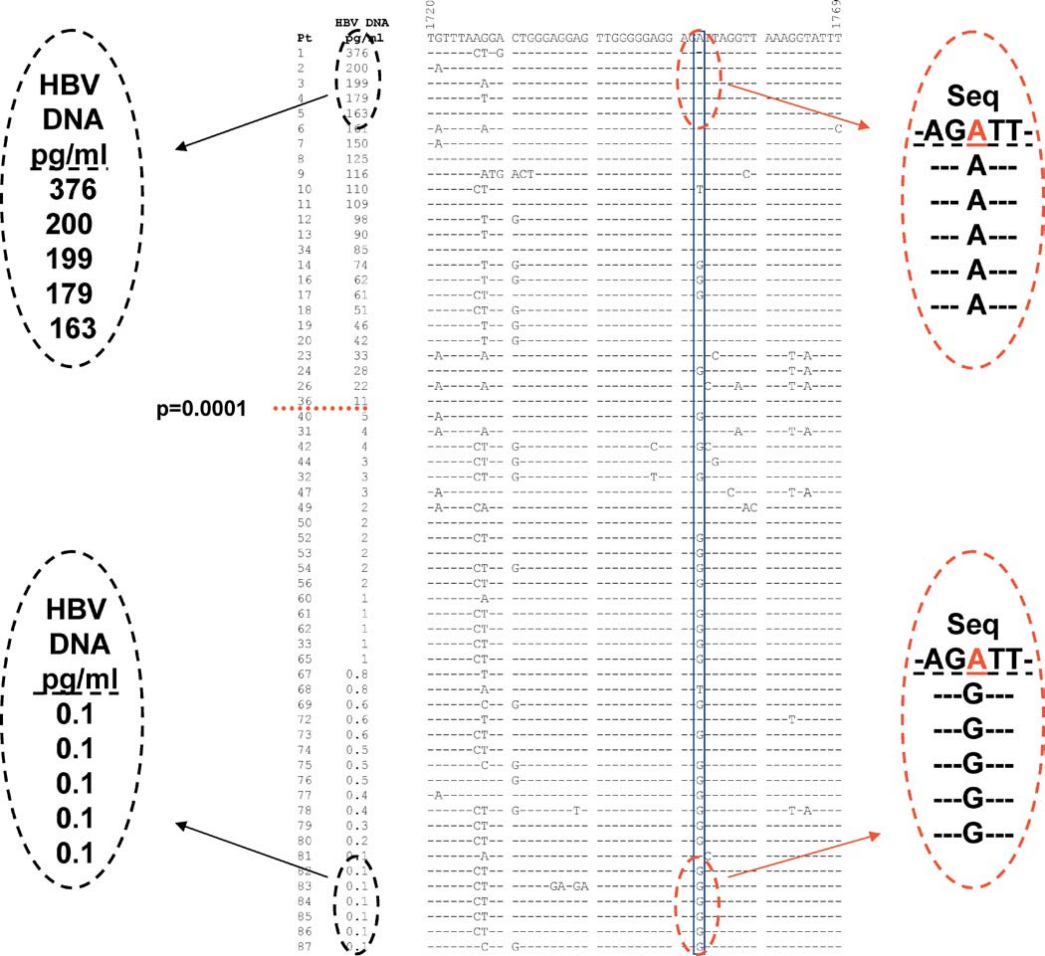
Distinct Segregation between High and Low Viremic HBV Individuals Is Correlated to Changes at Nucleotide Position 1752 Comparison of DNA sequences from nucleotide 1720–1769 with the HBV DNA concentration levels of the participants are illustrated. Sera was collected from 60 participants; DNA was isolated from sera and amplified with two rounds of PCR. Results of the sequences were aligned and compared.

To study the influence of HBV variants on the resultant dynamics of viremia in HBV carriers, we analyzed serum samples from 60 carriers for viral load using a commercially available test kit (Digene). The serum viral load among HBV carriers (Figure 1) was found to vary by more than three orders of magnitude (0.1–376 pg/ml). About 60% (36/60) of the carriers had single-digit pg/ml (0.1–5 pg/ml) concentration of viral DNA, while 40% (24/60) had greater than 10 pg/ml (11–376 pg/ml) concentration (Figure 1). To determine if viral variants had any link with viral load in the carriers, we amplified HBV DNA by PCR from serum, sequenced, and aligned the amplified fragments. Interestingly, within the entire viral genome, we observed a unique nucleotide position that showed significant correlation (*p* = 0.0001) with viral load as defined by the two broadly partitioned groups of serum viral load described above (using 10 pg/ml as an arbitrary cut-off). As there was no particular physiological phenotype linked to a threshold HBV viral load, we also applied cut-off values of 20 pg/ml and 50 pg/ml; in both instances, the results were similar to the 10-pg/ml value (data not shown). Although the HBV genotypes were predominantly genotype C, we observed a distinct tendency for carriers with high levels of serum HBV DNA to possess an A nucleotide at position 1752 of the virus sequence, while those with low levels of serum HBV DNA tend to have a G nucleotide at this position. This natural variation at nucleotide position 1752 was found to be located at the HBV genome's Enh II region, which has been shown to be a specific regulator of HBV replication [8,9,11,19,20].

### Natural Mutants of HBV with Different Viral Replication Efficiencies

**Figure 2:**
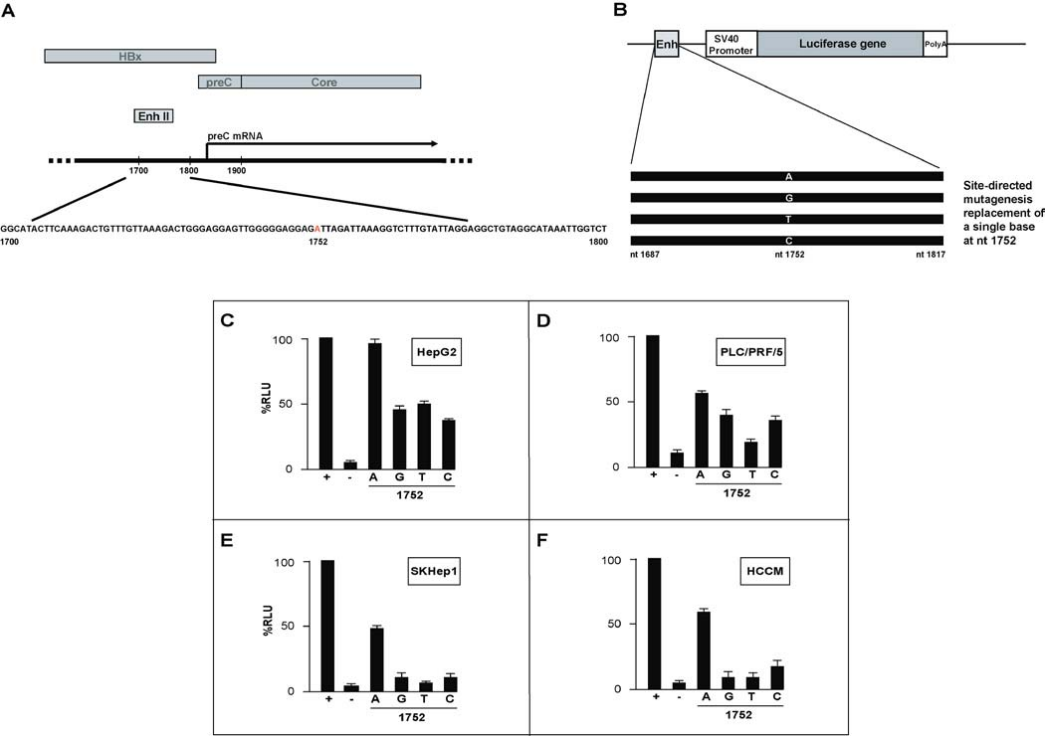
Effect of Nucleotide 1752 Base Substitution on Enh II Activity (A) The Enh II is located just upstream of the core promoter. The minimum sequence for enhancer activity has been previously defined at nucleotide 1687–1805 as shown. (B) Site-directed mutagenesis on nucleotide 1752 in the Enh II (1752A, 1752G, 1752T, and 1752C) and amplified fragments were inserted upstream of the SV40 promoter in an enhancerless luciferase reporter vector. (C–F) Four cell lines—HepG2, PLC/PRF/5, SKHep1, and HCCM, all derived from human hepatocellular carcinomas—were transfected with the respective Enh II clones (1752A, 1752G, 1752T, and 1752C). For each cell type, the first column (“+”) represents the luciferase activity of the internal positive control (promoter and enhancer). The second column (“−”) represents the activity of the internal negative control (promoter only). The other columns of each cell line represent the vectors with the promoter and the Enh II (nucleotide 1752A, 1752G, 1752T, or 1752C). Results of the luciferase assay were normalized to the level of the internal positive control (arbitrarily set at 100%).

As this variation resided in the viral enhancer element (Figure 2A), we proceeded to study the effects of this base substitution on transcriptional efficiency by cloning a 131-base-pair Enh II fragment bearing 1752A upstream of a SV40 promoter–luciferase reporter gene vector. Site-directed mutagenesis was then carried out to generate three other constructs bearing 1752G, 1752T, and 1752C so that four constructs differing from each other at only this single base nucleotide could be tested (Figure 2B). Transient transfections were carried out on the hepatic cell line HepG2 and subsequently assayed for luciferase activity. The results showed that the construct with 1752A strongly enhanced the SV40 promoter when linked to it in *cis,* resulting in higher levels of luciferase expression as compared to all other base substitutions (Figure 2C). As a positive control, the vector containing both the SV40 promoter and enhancer sequences resulting in optimal luciferase expression was used (“+” in Column 1 in Figure 2C). In addition, the cloning vector itself containing only the SV40 promoter–luciferase gene but without the enhancer element (“−” in Column 2 in Figure 2C) served as the negative control. To determine if these results were specific to only the host HepG2 cell line, we repeated the transfections in three other hepatic cell lines: SKHep1, PLC/PRF/5, and HCCM. The results were consistent with the initial results observed with HepG2 (Figure 2D, 2E, and 2F), further substantiating that the single base change at nucleotide position 1752 has a significant effect on the transcriptional efficiency of Enh II. These in vitro assays provide strong support for the effect of 1752A on higher HBV viral load, but it is worthwhile to note that this correlation is not absolute. As can be seen in Figure 1, the nucleotide change in 1752A cannot alone account for the wide variation in HBV viral load, suggesting that there may be additional contributing factors. Subsequent experiments were carried out using the HBV negative HepG2 cell line, as it has better growth characteristics than SKHep1, while PLC/PRF/5 and HCCM have integrated HBV genomes.

### Identification of Host Cellular Factor as hnRNP K

**Figure 3:**
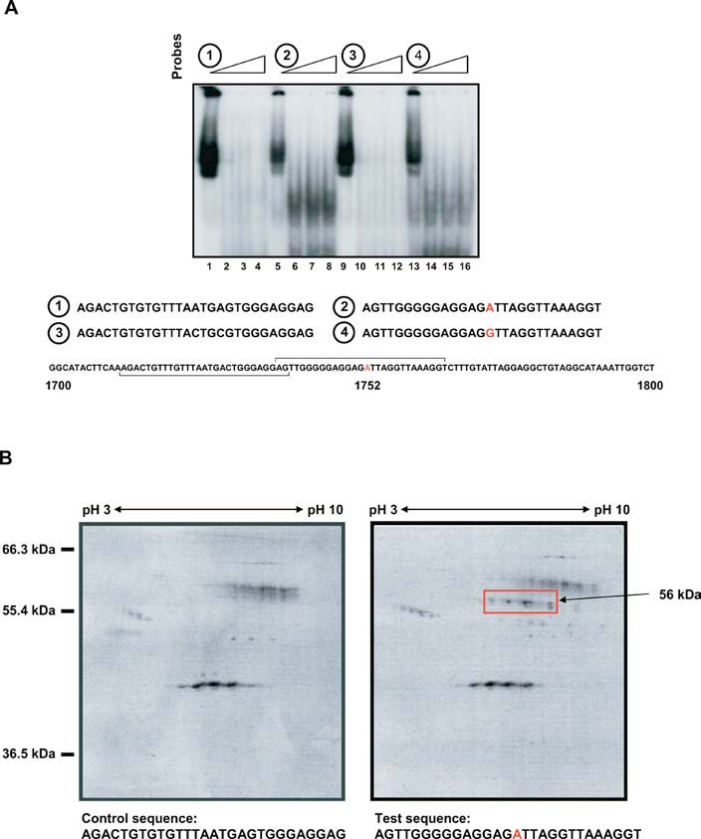
Evidence for the Involvement of a Host Cellular Protein in Enh II Activity and HBV Replication (A) Electrophoretic mobility shift assays were performed using HepG2 nuclear extracts with four different probes. Probe 1, lanes 1–4; probe 2 (1752A), lanes 5–8; probe 3, lanes 9–12; probe 4 (1752G), lanes 13–16. Each set of probes contains increasing concentrations (0.0 μg, 0.05 μg, 0.10 μg, and 0.15 μg) of non-specific competitor DNA [poly-(dI)-poly-(dC)], respectively. (B) 40 μg of nuclear protein extracts obtained from HepG2 cells was allowed to bind onto 5 mg Dynabeads M-280 streptavidin-biotin-oligonucleotides in the presence of 2:1 (w/w) ratio of non-specific competitor DNA poly (dI–dC). 1-D isoelectric focusing was followed by 2-D vertical separation on SDS-PAGE (10%). The estimated molecular weight of the specific protein spots detected by silver staining (arrow) is indicated.

In order to further establish and confirm the biological significance of this point mutation observed in the Enh II region, we wanted to demonstrate the presence of any direct physical HBV DNA–host interaction at the nucleotide position 1752 site. To this end, 28-mer oligonucleotide probes were designed to contain either the 1752A or 1752G nucleotide, with control probes taken from the immediate adjacent upstream sequence. Electrophoretic mobility shift assays were performed using HepG2 nuclear extracts with the respective probes, and the results showed a distinct DNA-binding protein was detected using the 1752A probe (Probe 2, lanes 5–8 in Figure 3A) along with a weaker band of similar size using the 1752G probe (Probe 4, lanes 13–16 in Figure 3A). Densitometric analysis of the bands indicated that the protein detected by Probe 2 was about 300% higher than that detected by Probe 4, suggesting that the 1752A probe has a higher binding affinity for the DNA-binding protein. These data lend support to the possibility that host DNA-binding proteins interact directly with the HBV viral sequence.

**Figure 4:**
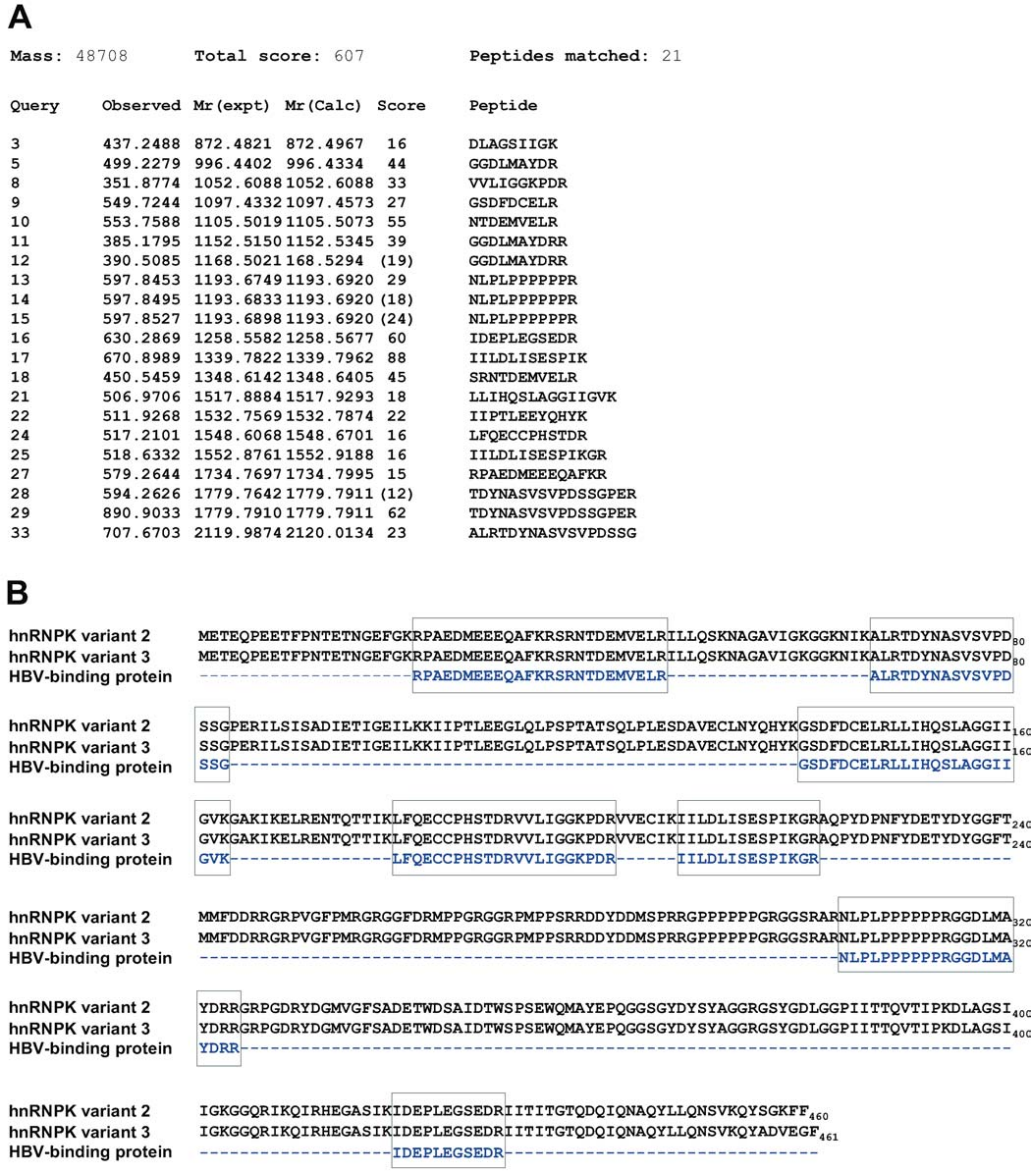
Identification of Host Cellular Protein as hnRNP K (A) Specific protein spots were cored out and de-stained, following which the gel plug was digested with trypsin. Sequence query of peptide fragments was carried out by Proteomic Research Services, using LC/MS/MS analysis. Results of the 21 sequenced peptides are illustrated. (B) Results of peptide sequencing of the 56-kDa protein by LC/MS/MS showed high homology scores to hnRNP K in sequence alignments.

To further characterize this DNA-binding protein, nuclear extract of HepG2 was passed through an affinity column tagged with the oligonucleotide probe (bearing 1752A). Bound material was eluted and subjected to 2-D gel analysis. Silver staining of the gels demonstrated positive enrichment of a specific DNA-binding protein compared with the non-specific binding control oligonucleotide probe (Figure 3B). The specific protein spots of approximately 56 kDa molecular weight were cored out and subjected to LC/MS/MS analysis (Figure 4A). Peptide sequencing results showed that 21 fragments of the unknown DNA-binding protein turned out to match perfectly with that of a known protein, hnRNP K (Figure 4B). The peptide sequence match and molecular weight of the predicted protein strongly suggest that the LC/MS/MS analysis has correctly identified the protein bound by the oligonucleotide-affinity column. The hnRNP K protein, which belongs to the family of hnRNPs, is about 56 kDa in size and has been shown to be involved in various cellular functions as described above.

### hnRNP K Is an Essential Modulator of Viral Replication

**Figure 5:**
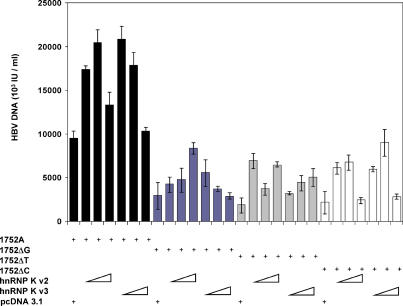
hnRNP K Is Involved in Modulating Viral Replication HepG2 cells were co-transfected with full-length replicative HBV clones (indicated by “+”) 1752A, 1752ΔG, 1752ΔT, and 1752ΔC (see Methods and Figure S1) with increasing dosages (50, 250, and 1,250 ng/μl) of hnRNP K variant 2 (v2) or variant 3 (v3) as indicated. pcDNA 3.1 serves as a control. Transfections were performed in duplicate; standard deviations are shown.

To demonstrate the functional connection of hnRNP K to the regulation of HBV viral load, a 1.4-kb RT-PCR fragment coding for the full-length hnRNP K gene from total RNA extracted from HepG2 cells was cloned into the mammalian expression vector pcDNA 3.1 (Invitrogen). There are two known variants of hnRNP K, termed “variant 2” and “variant 3,” which differ only at the last six amino acid residues of the C-terminal region. As there are no reported studies on the functional difference between the two variants, we decided to work with both variants. The two hnRNP K expression constructs were co-transfected separately into HepG2, together with a full-length 1752A replicative clone of HBV driven by the HBV core promoter rather than the CMV promoter [14,15] (see Figure S1) to determine the effects of hnRNP K on the replicative efficiency of the HBV construct. As predicted, the HBV viral concentration increased in a dose-dependent manner with hnRNP K concentration, with no significant functional difference between variants 2 and 3 (Figure 5). As a control, the empty expression vector pcDNA 3.1 did not have any effect on the HBV viral concentration. To further verify the impact of the single-base substitution at nucleotide position 1752, site-directed mutagenesis was carried out to generate three other full-length HBV replicative constructs bearing 1752ΔG, 1752ΔT, and 1752ΔC. Co-transfections with either hnRNP K variant 2 or variant 3 were performed as described above. All three constructs had 68–80% reduced HBV DNA when compared to the 1752A construct, indicating a lowered level of HBV viral load (Figure 5), although a high dosage of hnRNP K was able to augment the replication efficiency of the three constructs. Taken together, this set of co-transfection experiments provide definitive evidence that we have precisely mapped an important virus element and associated host component required for regulating HBV replication.

**Figure 6:**
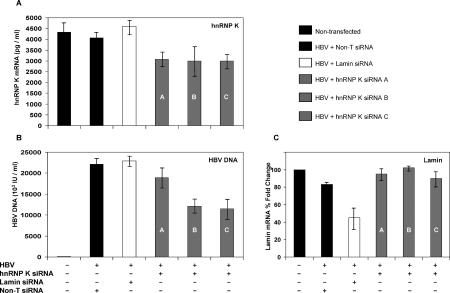
hnRNP K siRNAs Down-Regulate HBV Viral Replication (A) HepG2 cells were co-transfected with 1752A full-length replicative HBV clone either with or without hnRNP K siRNA (2 μg). Non-silencing (Non-T) and lamin A/C (Lamin) siRNAs were used as controls. hnRNP K expression was measured by quantitative real-time RT-PCR. (B) HBV viral load was quantitated by real-time PCR in cells transfected as described in (A). (C) Lamin A/C expression was measured from real-time RT-PCR. Ratios were normalized to 100% for the non-transfected cells. The results represent two independent samples; standard deviations are shown. Black columns represent either non-transfected cells or cells transfected with non-silencing siRNA. White columns represent cells co-transfected with HBV and lamin A/C siRNA. Grey columns represent cells co-transfected with HBV and hnRNP K siRNAs (A, Dharmacon; B, Qiagen; C, Proligo).

As a further critical proof of the regulatory role of hnRNP K in HBV viral load, the converse experiments to knock down hnRNP K should show a suppressive effect on viral load. To do this, siRNAs against hnRNP K were designed and obtained from three separate manufacturers. HepG2 cells were co-transfected with the replicative HBV full-length clone (1752A) together with the panel of hnRNP K siRNAs, and mRNA and DNA were analyzed 48 h after siRNA transfection. Non-silencing siRNA not matching to any genes and lamin A/C siRNAs were used as controls. The resultant hnRNP K mRNA levels as measured by quantitative real-time RT-PCR showed a 30% reduction relative to the non-transfected cells, non-silencing siRNA, and lamin A/C siRNA controls (Figure 6A). HBV viral loads were correspondingly decreased by 50% using both siRNAs from source B and C, while the siRNAs from source A achieved a 15% reduction (Figure 6B). The difference in effectiveness of siRNA from the three sources may be due to the different target regions that are selected to knock down hnRNP K, but the general suppressive trend was clear (see Methods). The lamin A/C mRNA levels measured by real-time RT-PCR in HepG2 cells transfected with lamin A/C siRNA showed a 45% reduction relative to the non-transfected cells, while non-silencing siRNA and hnRNP K siRNAs had no effect on the lamin A/C mRNA levels (Figure 6C). Taken together, these results confirm that hnRNP K plays a critical role in the process of HBV replication. The observation that changes in intracellular hnRNP K levels (both up and down) directly alter HBV replication efficiency points to a regulatory role for hnRNP K.

## Discussion

In this study, we were able to show that a host protein—hnRNP K—can be isolated by direct binding to a viral fragment derived from the HBV variant of the infected patient, and that hnRNP K binds to and modulates the replicative efficiency of HBV at a precise location at the Enh II regulatory region. Overexpression studies and RNA interference studies show a direct demonstration of the dependence of HBV on a host factor to modulate its replication efficiency, and hold promise as a new class of targets for the intervention of chronic hepatitis B infection.

The interesting observation that chronic HBV carriers have different serum viral loads spanning three logs (0.1–376 pg/ml in this study) between patients prompted us to investigate the possible correlation with viral genotype. While no such particular relationship emerged, we detected a solitary yet distinct natural mutation at nucleotide position 1752 that had an “A” that segregated with viral loads greater than 10 pg/ml, while those with “G” segregated with the samples of lower than 10 pg/ml. Of the 60 HBV genomes sequenced, only one sample showed a “T” at position 1752, while “A” and “G” variants were present in almost equal proportions. No natural “C” variant was seen, but this could be due to our limited sample size or an effect related to geographical distribution patterns.

To understand whether the natural variants at 1752 had any functional impact, we developed reporter constructs and tested all four possible 1752 variants for the ability to drive reporter gene transcription. It was evident that 1752A had higher activity than the three other non-A constructs. To investigate the underlying basis for this enhanced transcriptional activity, we proposed to search for evidence of possible physical interaction with DNA-binding proteins. Using an initial electrophoretic mobility shift assay followed by an affinity pull-down assay run on 2-D gel analysis, a 1752A oligonucleotide-affinity fragment was able to enrich a host binding factor sufficient for protein sequencing. The binding factor turned out to be hnRNP K, a known protein that has been shown to be involved in a number of cellular functions [12,13]. It has multiple modular domains, such as the K homology domains [21] and RGG boxes that allow it to interact with both DNA and RNA [22–25]. Reports have indicated that interactions between hnRNP K and single-stranded DNA are mediated by three K homology domains [21], and that hnRNP K exhibits specific binding and transactivation within the c-*myc* promoter [26–30]. hnRNP K was also shown to interact physically with the proto-oncogenes c-*src* and *vav* [22,24,31,32], thus allowing it to form multienzyme complexes and facilitate kinase cross-talk. This result suggests that hnRNP K is a versatile molecule that can act as a regulator of signal transduction and gene expression**.**

An important next step was to show that hnRNP K acts on a full-length HBV genome and not just the 131-bp enhancer fragment that was tested earlier in the luciferase reporter assay. To this end, we constructed four full-length replicative HBV clones, which were identical except for a single base change at position 1752. Each replicative clone variant was co-transfected with two different hnRNP K expression constructs that represent the two known hnRNP K variants, differing only at the C-terminal end of six amino acids. The results once again confirmed that the 1752A was more efficient than the other three variants, while there was no significant difference between either variant of hnRNP K in enhancing viral load. With escalating doses of hnRNP K, the viral load of the 1752G, 1752T, and 1752C variants increased in a dose-dependent manner. This suggests that the increased cellular concentrations of hnRNP K compensated to some degree for the reduced affinity bought about by the nucleotide substitutions.

In order to further demonstrate the role of hnRNP K in HBV replication, we wanted to test not only over-expression but also whether down-regulation of the cellular protein could have an effect. siRNAs designed to knock down endogenous hnRNP K were able to suppress both hnRNP K mRNA, and, along with it, the HBV viral load was greatly reduced. More importantly, the inclusion of control siRNA to lamin A/C suppressed lamin A/C mRNA but had no effect on HBV viral load, thus strengthening the link between hnRNP K and HBV.

The mechanistic aspect of hnRNP K on HBV replication needs to be further explored, and efforts are currently under way. There are possibilities such as hnRNP K polymorphisms [33] (see Figure S2), phosphorylation states [21,22], transcriptional regulation, and perhaps other cellular protein factors that may act in concert to support the replication activity of HBV in the host. That hnRNP K binding is mapped precisely to a single-base polymorphism in the HBV genome's regulatory region suggests that a dynamic interaction exists between specific natural mutants of the HBV Enh II region and the patient's genotype composition that together function to determine the overall outcome of viral replication efficiency. Indeed, a deeper understanding of how this works will provide insights into defining virulence and fitness of a virus.

Our study shows that the overall viral replication efficiency is determined by a combination of both viral sequence and interaction with specific host proteins. While development of antivirals is an established path, targeting the host remains surprisingly unexplored. Interestingly, a recent study on anti-EGFR antibody treatment of breast cancer cells showed a decrease in the cell-replication rate with a corresponding reduction in hnRNP K expression levels [34]. This suggests that hnRNP K levels can be modulated by anti-EGFR treatment and holds possibilities for new indications for existing and approved pharmaceuticals as an approach to alter HBV viral load in chronic carriers. Overcoming entrenched, chronic viral infections is not a straightforward solution and will eventually involve a combination therapy of targeting the virus directly, blocking host support proteins, and simultaneously employing immuno-modulating agents to bring about long-term viral clearance.

## Supporting Information

Figure S1 Construction of the Full-Length HBV Replicative Clone (53 KB PDF)

Figure S2 SNPs in the hnRNP K Gene from 18 Volunteers Compared with SNPs Extracted from Ensembl and Celera Databases (59 KB PDF)

**Patient Summary**

**Background**

Hepatitis B is a virus that can cause long-term health problems, including cirrhosis of the liver and liver cancer in people who are not able to clear the virus from their body. Although some drugs can suppress the multiplication of the virus, these drugs do not cure the patient of hepatitis B. Another way of tackling the virus might become clear if it were known exactly how the virus interacts with the patient's cells.

**What Did the Researchers Do?**

They noticed that among patients who carried the hepatitis B virus (HBV), some had higher amounts of the virus in their blood compared to others. The patients with higher levels tended to have a virus that had one particular DNA type, and this particular virus type could bind more closely to a protein found in humans: hnRNP K. In the laboratory, when the investigators increased the amount of this protein in cells infected with HBV, the virus multiplied more; when they decreased it, the virus multiplied less.

**What Does This Mean?**

This work suggests that one way to control the multiplication of HBV in infected people is indirectly, by altering levels of the protein hnRNP K. The next step will be to identify ways to do this safely and reliably.

**Where Can I Get More Information?**

Medline Plus has a section on hepatitis B:

http://www.nlm.nih.gov/medlineplus/ency/article/000279.htm

The World Health Organization has a set of information on hepatitis, including hepatitis B:

http://www.who.int/topics/hepatitis/en/

## Abbreviations

DTT: dithiothreitol
EDTA: ethylene diamine tetra-acetic acid
HBV: hepatitis B virus
HEPES: *N*-2-hydroxyethylpiperazine-*N′*-ethane sulfonic acid
hnRNP: heterogeneous nuclear ribonucleoprotein
LC/MS/MS: liquid chromatography/tandem mass spectrometry
siRNA: small interfering RNA
