# Supplementary material for: Host Heterogeneous Ribonucleoprotein K (hnRNP K) as a Potential Target to Suppress Hepatitis B Virus Replication
Source: PLoS Med. 2005 Jul 26;2(7):e163. doi: 10.1371/journal.pmed.0020163 (PMC1181871; doi:10.1371/journal.pmed.0020163)
Supplement: Figure S1 — (53 KB PDF) [file pmed.0020163.sg001.pdf]

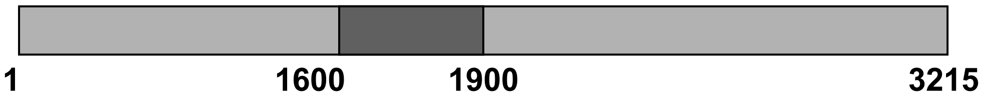

ATCC clone available in pBR325. EcoRI site at both ends. Primers designed to amplify 2 fragments: 1-1900 and 1600-3215. Ligation using internal EcoRI (1/3215) ensures continuous viral ORFs.

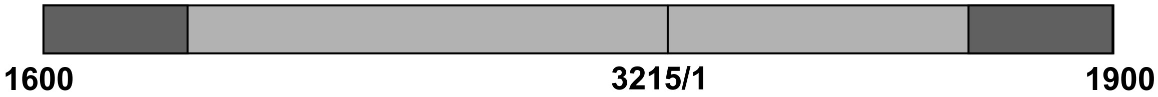

Replicative clone with promoter (1600-1900) at its 5' end and termination region (1600-1900) at its 3' end.
The construct was cloned into NruI site in pcDNA3.1. Viral transcription is under its own promoter, as NruI is outside the pCMV promoter.

Supporting Information Figure S1. Description of cloning of the full-length replication HBV construct.

Ng *et al*., 2005
