# Supplementary material for: Host Heterogeneous Ribonucleoprotein K (hnRNP K) as a Potential Target to Suppress Hepatitis B Virus Replication
Source: PLoS Med. 2005 Jul 26;2(7):e163. doi: 10.1371/journal.pmed.0020163 (PMC1181871; doi:10.1371/journal.pmed.0020163)
Supplement: Figure S2 — (59 KB PDF) [file pmed.0020163.sg002.pdf]

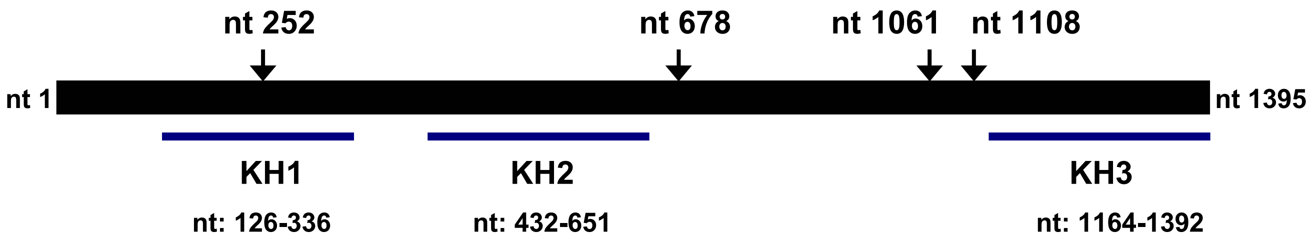

Location (nt)	AA change	Frequency	Source
252C>T	Silent	2 samples	this study
678C>T	Pro>Ser	not validated	dbSNP:11548847
1061A>C	Pro>Gln	not validated	dbSNP:11548850
1108 to 1122	Deletion	2 samples	this study

Supporting Information Figure S2. SNPs (single nucleotide polymorphisms) in the hnRNP K gene were extracted from Ensembl (www.ensembl.org) and Celera databases. In addition, we cloned and sequenced the hnRNP K full length cDNA from 18 normal volunteers. Only 2 SNPs were reported from the dbSNP and both have not been validated. We have found a novel SNP at nt252 that involves a C>T change, but is non-synonymous. An interesting deletion was observed in 2 different samples in which a 15 base deletion was found just upstream of the KH3 domain (nt1108 to nt1122, nt: ATGATTATTCCTATG). There are a number of SNPs in the UTR (untranslated region) and intronic regions reported in the public databases, but none have been validated (results not shown).

Ng *et al.*, 2005
